# A Microfluidic Strategy to Capture Antigen‐Specific High‐Affinity B Cells

**DOI:** 10.1002/anbr.202300101

**Published:** 2024-04-26

**Authors:** Ahmed M. Alhassan, Venktesh S. Shirure, Jean Luo, Bryan B. Nguyen, Zachary A. Rollins, Bhupinder S. Shergill, Xiangdong Zhu, Nicole Baumgarth, Steven C. George

**Affiliations:** ^1^ Department of Biomedical Engineering University of California Davis CA 95616 USA; ^2^ Department of Pathology, Microbiology, and Immunology University of California Davis CA 95616 USA; ^3^ Department of Physics and Astronomy University of California Davis CA 95616 USA; ^4^ Department of Molecular Microbiology and Immunology Bloomberg School of Public Health and Department of Molecular and Comparative Pathobiology School of Medicine Johns Hopkins University Baltimore MD 21205 USA

**Keywords:** affinities, avidities, cell separations, immunology

## Abstract

Assessing B cell affinity to pathogen‐specific antigens prior to or following exposure could facilitate the assessment of immune status. Current standard tools to assess antigen‐specific B cell responses focus on equilibrium binding of the secreted antibody in serum. These methods are costly, time‐consuming, and assess antibody affinity under zero force. Recent findings indicate that force may influence BCR‐antigen binding interactions and thus immune status. Herein, a simple laminar flow microfluidic chamber in which the antigen (hemagglutinin of influenza A) is bound to the chamber surface to assess antigen‐specific BCR binding affinity of five hemagglutinin‐specific hybridomas from 65 to 650 pN force range is designed. The results demonstrate that both increasing shear force and bound lifetime can be used to enrich antigen‐specific high‐affinity B cells. The affinity of the membrane‐bound BCR in the flow chamber correlates well with the affinity of the matched antibodies measured in solution. These findings demonstrate that a microfluidic strategy can rapidly assess BCR‐antigen‐binding properties and identify antigen‐specific high‐affinity B cells. This strategy has the potential to both assess functional immune status from peripheral B cells and be a cost‐effective way of identifying individual B cells as antibody sources for a range of clinical applications.

## Graphical abstract

This study demonstrates that a simple microfluidic strategy can rapidly assess BCR‐antigen binding properties and identify antigen‐specific high‐affinity B cells. This strategy has the potential to both assess functional immune status from peripheral B cells and be a cost‐effective way of identifying individual B cells as antibody sources for a range of clinical applications.

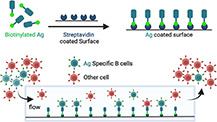

## 1 Introduction

Activation, clonal expansion, and affinity maturation of B cells in germinal centers are considered the hallmarks of adaptive immunity, which are triggered when challenged by foreign antigens (e.g., viral or bacterial infection).^[^ ^1^ ^]^ Upon rechallenge, memory B cells (B_mem_) differentiate rapidly into plasma cells which then secrete antigen‐specific high‐affinity antibodies to facilitate rapid pathogen clearance.^[^ ^2^ ^]^ The serum antibody pool primarily reflects the antibody‐producing plasma cell population and thus does not fully capture the dynamic characteristics and immunogenic potential of antigen‐specific B_mem_, which are known to arise earlier from germinal center responses than plasma cells and thus carry overall fewer mutations.^[^ ^3^ ^]^

The ability to precisely measure B cell–antigen interaction strength through the membrane‐bound B cell receptor (BCR), particularly in B_mem_, would greatly enhance the evaluation of functional immunity (i.e., the immune status of an individual following infection or vaccination) as it may better correlate with immune protection.^[^ ^4^ ^]^ Moreover, the ability to isolate antigen‐specific B cells with known antigen‐binding avidities could aid in rapid identification and creation of monoclonal antibody‐based therapeutics.

Assessment of binding between the membrane‐bound BCR or the secreted antibody can be performed using various methods. For example, enzyme‐linked immunosorbent spot or flow cytometry allows for the measurement of B cell activation or BCR expression, respectively, at single‐cell resolution following BCR–antigen engagement. However, these methods do not allow isolation of the cells. In contrast, fluorescence‐activated cell sorting (FACS)^[^ 5, 6, 7 ^]^ or droplet‐based microfluidics^[^ 8, 9 ^]^ can be used to isolate antigen‐binding B cells. However, these cell‐separation methods cannot characterize binding properties of the BCR, such as avidity or on‐ and off‐rates. Furthermore, these methods do not control the force exerted on the bond between the BCR and antigen. More specifically, the fluorescent‐conjugated antigens are incubated with the cells, allowed to come to equilibrium under static conditions with either the membrane‐bound BCR or the secreted antibody (droplet‐based microfluidics^[^ 8, 9 ^]^), and the fluorescence is used to separate the cells. In vivo, B cells interrogate antigens with the BCR under force, and naïve B cells are strongly activated when this force exceeds 50 pN.^[^ ^10^ ^]^ This force may modify a range of B cell responses, including activation and antigen internalization.^[^ ^11^ ^]^

Overall B cell binding avidity (sometimes referred to as “effective affinity”) depends on both epitope density and the intrinsic affinity of the BCR to the cognate antigen. While BCR binding affinity is generally acknowledged to be the primary determinant of B cell activation and recruitment in vivo and thus as prognostic of immune protection,^[^ 12, 13 ^]^ B cells recruited to the germinal center generally encounter the same epitope density, and thus intrinsic affinity of the BCR is a useful surrogate. Nevertheless, it is crucial to recognize that antibodies with similar affinity (ratio of kinetic on‐rate and off‐rate) can have on‐ and off‐rates that vary over four orders of magnitude.^[^ ^14^ ^]^ The kinetic on‐ and off‐rates themselves can profoundly impact B cell biology. Indeed, antibody maturation and selection, at least in some cases, has been attributed to enhancement of the kinetic on‐rate.^[^ 15, 16 ^]^ While established techniques like surface plasmon resonance (SPR) are available for characterizing the kinetic properties (on‐rate and off rates) of antibodies, it's important to note that the B_mem_ themselves do not secrete antibodies. Instead, they first need to differentiate into antibody‐secreting plasma cells. This distinction underscores an unmet need for characterizing the binding properties of membrane‐bound BCR.

Utilizing a simple microfluidic strategy to control shear force and antigen presentation, we have developed a method to simultaneously capture and enrich antigen‐specific high‐affinity B cells and quantify force‐dependent B cell binding avidity and kinetic properties for a pool of B cells. Our results demonstrate that 1) both (shear) force and bound lifetime can be used to enrich a population of antigen‐specific high‐affinity B cells; and 2) the avidity constants of the B cells measured in the device correlate well with the affinity of the secreted antibodies measured in solution.

## 2 Experimental Section

### 2.1 Mice

C57BL/6 mice (The Jackson Labs) and “SwHEL” BCR transgenic mice expressing a BCR specific for hen egg lysosome (HEL)^[^ ^17^ ^]^ were provided with food and water at libitum and held under SPF housing conditions at the University of California (UC) Davis. Breeding pairs for SwHEL mice were obtained from Dr. Roger Sciammas (UC Davis) with kind permission from Dr. Robert Brink (Garvan Institute of Medical Research, New South Wales, Australia). Male and female mice, age 8–15 weeks, were used as the source of HEL‐specific B cells. All experiments involving mice were conducted in strict adherence to protocols approved by the UC Davis Animal Care and Use Committee.

### 2.2 Hybridomas

To assess adherence of influenza hemagglutinin‐specific B cells, we assessed five previously characterized hybridoma cell lines generated from influenza A/8/34 immunized BALB/c mice. As a negative control hybridoma, we used DS.1, specific to IgMa (**Table** **1**). Cells were grown in RPMI 1640 containing 10% FBS and 1:100 P S^−1^ (Gibco 15 140 122). Cells were collected when they were about 70% confluent and looked round, smooth, and quite large.

**Table 1:** Specificity and subtype of hybridoma lines used in experiments

Hybridoma Line	Specificity	IgG subtype/Idiotype
H37‐41‐1 (H37)	Hemagglutinin (HA)1 of influenza A/PR/8/34	IgG1/C4
H35‐C12.6.2 (H35)	Hemagglutinin (HA)1 of influenza A/PR/8/34	IgG2a/C12
H36‐4.5.2 (H36)	Hemagglutinin (HA)1 of influenza A/PR/8/34	IgG2a/other
H163‐130F2‐2 (H163)	Hemagglutinin (HA)1 of influenza A/PR/8/34	IgG2a/C12
H143‐16A8‐6 (H143)	Hemagglutinin (HA)1 of influenza A/PR/8/34	IgG2a/C12
DS.1	Mouse IgMa	IgG1/other

### 2.3 B Cell Isolation

For binding studies involving primary B cells, cells were obtained from spleens of SwHEL or wild‐type (WT) C57BL/6 mice. All experiments outlined using tissues from mice were done following approval of protocols (N. Baumgarth) by the UC Davis Animal Care and Use Committee. Single‐cell suspensions were generated by grinding spleens between the frosted ends of two glass slides and filtered through a 70 mm nylon mesh. All samples were then treated with ACK lysis buffer,^[^ ^18^ ^]^ refiltered through nylon mesh, and resuspended in RPMI or staining media. Single‐cell suspensions were counted and blocked with anti‐FcγR (mAb 2.4.G2). Then cells were labeled with biotinylated HEL generated in‐house and antimouse CD19‐CF594 (ID3, Biolegend), followed by staining with streptavidin–allophycocyanin and live/dead Aqua (Thermo Fisher) to assess frequencies of HEL‐binding cells by flow cytometry, which were about 20–25% of total cells.

### 2.4 Flow Cytometry

For staining of surface Ig, hybridoma cells were collected, washed, and resuspended in staining buffer. Cells were blocked with anti‐FcgR (2.4.G2), surface stained with APC‐Cy7 antimouse Ig kappa light chain (BD 561 353), and then stained with Live/Dead Aqua (Thermo Fisher, L34966). Each step was done for 15 min on ice followed by washing the cells in staining buffer. Cells were analyzed using a BD FACS Symphony flow cytometer. Data analysis was done using FLowJo software.

### 2.5 Purification of Monoclonal Antibodies

Hybridoma supernatant was collected 3–5 days after cells were seeded into flasks when medium turned from pink to orange and filtered through a 0.22 μm filter followed by ammonium sulfate precipitation using ammonium sulfate salt, added slowly (from 313.5 g to 1000 mL) to reach ≈50% saturation and then incubated for 5–15 h at 4 °C. Supernatants were centrifuged at 5000 g for 30 min at 4 °C. The pellet was resuspended in PBS and then dialyzed against at least three changes of PBS for 24–48 h. IgG was purified by low‐pressure, HiTrap Protein G column chromatography following the manufacturer's instructions (Cytica HiTrap Protein G HP, 17 040 501). After elution, antibody was concentrated and buffer exchanged into PBS or storage buffer (10 mm Tris, 150 mm NaCl, 0.1% NaN3, pH 8.2). The protein concentration was determined by measuring the optical density at 280 nm. For IgG, an absorption of OD_280_ of 1.35 was set to equal 1 mg mL^−1^ IgG.

### 2.6 ELISA

Influenza virus‐specific enzyme‐linked immunoassay (ELISA) was performed as described.^[^ ^18^ ^]^ Briefly, ELISA plates (MaxiSorp 96 well plates, Thermo Fisher #12‐565‐135) were coated overnight at room temperature with influenza A/Puerto Rico/8/34 virus particles (400 HAU mL^−1^; in house) purified from the allantoic fluid of infected day 14 embryonated hen eggs, precipitated with polyethylene glycol and purified via a sucrose gradient centrifugation. Plates were washed and nonspecific binding was blocked with 1% newborn calf serum, 0.1% dried milk powder, and 0.05% Tween 20 in PBS (ELISA blocking buffer). Following pilot studies, all HA‐specific mAb were added to the plate at a starting dilution of 100 ng mL^−1^ (except H143‐16A8‐6 which was used at 10 μg mL^−1^) and then were serially diluted by twofold increment in PBS. Binding was revealed with biotinylated anti‐IgG (Southern Biotech 1030‐08), followed by streptavidin horseradish peroxidase (Vector SA‐5004) both diluted in ELISA blocking buffer (0.005% 3,39,5,59‐tetramethylbenzidine in 0.05 M citric acid buffer, PH 4.0 and 0.015% hydrogen peroxide (Spectrum H1070). The reaction was stopped with 1 N sulfuric acid after 20 min. Absorbance was measured at 450 nm (595 nm reference wavelength) on a spectrophotometer (SpectraMax M5, Molecular Devices).

### 2.7 Microfabrication and Device Surface Preparation

Microfluidic devices were prepared using standard methods of soft lithography.^[^ 19, 20 ^]^ In brief, a SU8 master mold was prepared and the microdevice was created by casting polydimethylsiloxane (PDMS; Dow Corning, Midland, MI) on the SU‐8 master mold. Once, polymerized, the PDMS was peeled off the master mold. Glass slides (Thermo Fisher Scientific) were rinsed in purified water and were then plasma bonded to the PDMS to form channels of 10 mm × 0.8 mm × 0.1 mm (length × width × height). The simple device design of a single long rectangular channel is a strength as it should be easily translated to other labs. The novelty of our method lies in the capture, enrichment, and characterization of B cells based on the binding avidity of the membrane‐bound BCR under controlled force.

The microfluidic devices were coated with the desired antigens following well‐documented streptavidin‐biotin chemistry.^[^ ^21^ ^]^ The devices were first treated with plasma for 45 s and immediately used for coating. In brief, the devices were incubated at room temperature with (3‐Mercaptopropyl)trimethoxysilane (3.65% in absolute ethanol, Sigma‐Aldrich) for 1 h, washed twice with absolute ethanol, then incubated with N‐γ‐maleimidobutyryl‐oxysuccinimide ester (1 mm in absolute ethanol, Thermo Fisher Scientific) for 30 min, washed twice with absolute ethanol, and finally incubated with NeutrAvidin at 4 °C (100 ug mL^−1^ in PBS, Thermo Fisher Scientific) for 2 days and washed twice with PBS. The devices were then incubated at 4 °C with the biotinylated antigen prepared in PBS at a desired concentration for 2 days, washed twice with PBS, and incubated with BSA (10 mg mL^−1^ in PBS) for 15 min before perfusing the cells.

### 2.8 Cell Perfusion Through the Microfluidic Device

We used a pipette tip (200 μL) connected at the inlet as the entry port for cells and fluid, and the outlet of the microfluidic chip was connected by Tygon tubing to a high‐precision syringe pump (Harvard Apparatus), operated in withdraw mode. The devices were first equilibrated by perfusing PBS for 2 min at 200 μL min^−1^. The devices were placed on an IX83 inverted microscope (Olympus) equipped with a high‐speed camera and hardware to acquire stream acquisitions.

Cells suspended at a prescribed concentration (generally 300 000 cells mL^−1^) were added to the source tip after setting desired flow. The concentration of cells was high enough to allow for a significant number of binding events to occur while low enough to allow for individual cells to probe the surface as individual events (i.e., neighboring cells did not interact with each other). The devices were imaged using a bright‐field, 10× objective and an image acquisition speed of 8 frames per second. Four different flows were used (10, 20, 50, and 100 μL h^−1^) corresponding to wall shear stress of 0.03, 0,06, 0,15, and 0.3 dynes cm^−2^ or tensile stress of the bond of ≈65, 130, 325, and 650 pN.^[^ ^22^ ^]^ For higher flows (50 and 100 μL h^−1^), the acquisition speed was increased to 30 frames s^−1^, and, to accommodate for this higher acquisition speed, total acquisition time was reduced to 2 min. The images were saved for later analysis.

In some experiments, the hybridoma cell lines were labeled with either CellTracker BMQC (violet), CMFDA (green), CMMTR (Orange), or Deep Red. A mixture of differently labelled hybridoma cells at 1.5 × 10^6^ cells mL^−1^ was perfused through the device. The devices were imaged using a FV1200 Fluoview confocal laser scanning microscope (Olympus) connected to FV10‐ASW image acquisition and analysis software (Olympus). The confocal microscope was used for simultaneous time lapse recording of four color channels. The images were acquired at the maximum allowable acquisition rate of ≈1 frame s^−1^.

### 2.9 Analysis of Cell Binding in the Device

Each cell perfusion experiment was analyzed to find: 1) total number of cells flowing across the field of view; 2) the number of cells bound to the surface; and 3) the time each cell remained bound (bound lifetime). To find these, the microscopy‐obtained time lapse image sequences were analyzed using TrackMate (an ImageJ plugin) following the recommended protocol.^[^ ^23^ ^]^ The software recorded cell positions from each image in a time sequence. The algorithm then generated tracks of cell movements using consecutive images from the sequence. The software generated images with tracks that were color coded by the order in which the cells entered the field of view. The “tracks” and “spots” data files generated from TrackMate were exported to the receptor‐ligand nonequilibrium kinetics^[^ ^24^ ^]^ to compute capture efficiency and bound lifetimes. Capture efficiency is defined as the ratio of the number of bound cells (*N*_b_) to the total number of cells (*N*_0_) flowing across the field of view. A binding event was defined as a cell moving less than 0.5 μm. The minimum binding time criterion (i.e., cells remain bound for greater than this time) was chosen as 10, 20, 50, or 100 s. In a small number of cases where a mixture of cells was perfused, the acquisition speed was <5 frames s^−1^ and an accurate count with the automated analysis was not possible; in these cases, the analysis was performed manually.

#### 2.9.1 Mathematical Model of Cell Perfusion Through the Microfluidic Device

A mathematical model capable of simulating laminar fluid flow and cell movement through the device was created and solved (finite‐element) using COMSOL Multiphysics 5.2a software. The computer‐aided design file of the device with all three dimensions was imported as the geometry of the device. The laminar flow module was used to drive flow through the device at a desired fluid flow rate. The no‐slip boundary condition was applied for all surfaces except the microfluidic entrance and exit. To find the cell trajectories, particle tracing module was used. Particle properties were set as typical cell properties (radius = 5 μm; *r* = 1.086 g cc^−1^). The cells entering the device through the inlet and their initial position at the inlet boundary were set at randomly chosen locations. The model was set so that cells entered the device in a short pulse of 1 s and at every 10 s interval thereafter. The number of cells entering every 10 s with each pulse were computed using the known perfusion concentration of the cells (300 000 cells mL^−1^) and *Q* = 10, 20, 50, 100, 200 μL h^−1^. Because of gravity, the cells eventually settled in the device. They also experienced fluid drag under the laminar fluid flow, which moved them along the length of the device. The cells that first contacted the bottom surface of the device and the cells exiting the device were frozen at those boundaries to create a visual demonstration of the simulations.

### 2.10 Estimation of Effective *k*_on_ and *k*_off_

Assuming cell binding to the substrate as a first‐order process, the number of bound cells should obey the following rate law: d*N*_b(1)_/d*τ* = *k*_on_(*N*_0_−*N*_b(1)_), where *τ* is time that the cell needs to pass through the field of view or residence time of cells flowing across the field of view, *N*_b(1)_ is the number of cells that bind to form the initial tether (or bind at least 1 s), and *N*_0_ is the total number of cells that enter the field of view per *τ*. The analytical solution to the above equation is: *k*_on_ = (−ln((*N*_0_−*N*_b(1)_)/*N*_0_)/*τ*). We performed simple linear regression on the data to determine *k*_on_.^[^ ^25^ ^]^ Thus, *k*_on_ determined in our assay is a function of number and diffusivity of BCR/ligands on the substrate and the intrinsic single molecule on‐rate.

A bound cell detaches from the substrate due to kinetic off‐rates. We assumed a first‐order process for cell detachment: $dN_{b}/dt_{b}=k_{\text{off}}N_{b}$, where *N*_b_ is the number of bound cells for at least time *t*_b_. The analytical solution for this equation is *k*_off_ =  ln(*N*_b(tb)_/*N*_b(10)_)/(*t*_b_−10) where *N*_b(tb)_ and *N*_b(10)_ are the number of cells that bind at least *t*_b_ and 10 s, respectively, Thus, the off‐rate is simply the slope of the line graph between fraction of bound cells and their bound lifetime, which is also referred to as survival curves.^[^ 26, 27 ^]^ Data fits were weighted by the fraction of bound cells. The force‐dependent *k*_off_ is related to force independent $k_{\text{off}}^{0}$ by the Bell model,^[^ ^28^ ^]^ $k_{\text{off }}=k_{\text{off}}^{0}e^{x_{\beta }F/K_{B}T}$, where *x*_*β*_ is reactive compliance of the intermolecular bond, *F* is the force applied on the bond, and *K*_B_ is Boltzmann's constant. In the present study, *F* was varied by applying fluid shear force (*F* can be calculated from the wall shear stress as detailed previously^[^ ^24^ ^]^) and linear regression was performed on log transformed data to determine $k_{\text{off}}^{0}$.

### 2.11 Oblique‐Incidence Reflectivity Difference

Five purified anti‐HA IgG molecules, H35‐c12.6.2, H36‐4.5.2, H37‐41‐1, H143‐12, and H163‐12‐2 (Table 1) were separately diluted with 1 × PBS to printing concentration of 6.7 μm (i.e. 1 mg mL^−1^). Bovine serum albumin (BSA) and biotinylated bovine serum albumin (BBSA) were diluted separately with 1 × PBS to printing concentration of 3.8 μm (i.e. 0.25 mg mL^−1^). On an epoxide‐functionalized glass slide (1″ × 3″), we printed six identical microarrays from these eight printing solutions. Each array consisted of 39 replicates of 5 IgG molecules and BSA, and three replicates of BBSA. BSA and BBSA were negative and positive controls, respectively. Each microarray was housed in a separate, optically accessible reaction chamber (12 mm × 6 mm ×0.4 mm; i.e., volume = 29 μL). Before binding assays were conducted, the microarrays were washed with 1 × PBS, blocked with a solution of BSA at 2 mg mL^−1^ in 1 × PBS for 30 min, and then washed again in 1 × PBS.

For affinity binding assays, we prepared 300 nm solutions of influenza A/PR/8/34 virus H1N1 haemagglutinin (HA) recombinant antigen (or Rec‐HA; The Native Antigen Company, Kidlington, Oxfordshire, UK) in 1x PBS. For avidity binding assays, we prepared a 2 × 10^−4^ HAU/ml solution of influenza A/PR/8/34.

For the affinity binding reactions, we first replaced the 1x PBS in the reaction chamber with the HA solution and then incubated the microarray in the HA solution under a slow flow condition at 2.5 μL min^−1^ for 30 min for the association phase of the reaction. After the association phase, we replaced the HA solution in the chamber with 1x PBS and then incubated the microarray in 1 × PBS (under a slow flow condition at 20 μL min^−1^) for another 90 min for the dissociation phase of the reaction.

For avidity binding reaction, we replaced the 1x PBS in the reaction chamber with a solution of 2 × 10^−4^ HAU mL^−1^ A/PR8 and then incubated the microarray in this solution under a slow flow condition at 2.1 μL min^−1^ for 4 h for the association phase. After the association phase, we replaced the A/PR8 solution with 1x PBS and then incubated the microarray again under a slow flow condition at 10 μL min^−1^ for another 2 h for the dissociation phase of the reaction.

To measure binding curves during the reaction, we used an oblique‐incidence reflectivity difference (OI‐RD) scanner, described previously.^[^ 14, 29, 30, 31 ^]^ With this scanner, we measured the phase change in a reflected optical beam due to the presence of a biomolecular layer on a solid support during the reaction. Similar to an SPR sensor, the phase change detected with an OI‐RD scanner was converted to the surface mass density of the biomolecular layer. In the present work, the scanner measured in real time the amount of Rec‐HA or A/PR8 virions captured by printed (i.e., immobilized) IgG molecules and the control molecules. The association–dissociation curves (i.e., binding curves) were fit to yield rate constants *k*_on_ and dissociation rate constants *k*_off_. Before and after each reaction, we also acquired OI‐RD images for endpoint analysis.

### 2.12 Spleen Cell Labeling

SwHEL spleen cells were perfused in a device coated with HEL and then washed with PBS. Cells bound in the device were labeled with antimouse CD19 Alexa Fluor 647 and antimouse CD3 Alexa Fluor 488 antibodies. Cells in the device were imaged using a FV1200 Fluoview confocal laser scanning microscope (Olympus) connected to FV10‐ASW image acquisition and analysis software (Olympus).

### 2.13 Statistics

Statistical analysis and data fitting was performed using GraphPad Prism (10.1.1). Unless otherwise mentioned, data was presented as mean ± standard error of the mean (SEM) with individual data points (*n* ≥ 3) presented on the graphical charts. Statistical significance indicated *p* < 0.05 by one‐way analysis of variance. Multiple comparisons were performed using Tukey's multiple comparison test. The error bars on the parameters estimated by data fitting (Figure 3 and 6) were standard deviations and were determined using residuals and degrees of freedom of the regression model.

## 3 Results

**Figure 1:**
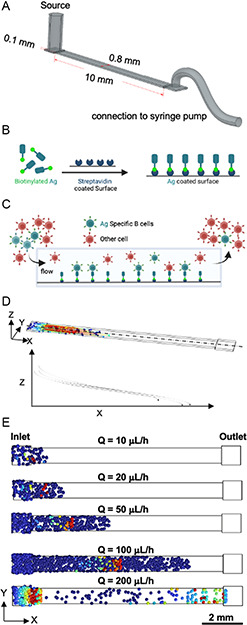
A) Schematic of antigen functionalized on the surface of the device and B) cells binding to the antigen‐coated surface. C) COMSOL Multiphysics model of cells entering the microfluidic device and flowing horizontally and D) settling to the bottom along the length of the device at different flow rates. Cells that contact the surface (blue) are frozen in place.

To examine BCR–antigen interactions under force, we utilized a simple single‐rectangular‐shaped microfluidic channel (**Figure** **1**A) formed by creating a groove (walls and ceiling of channel) of defined dimensions in PDMS and then bonding the PDMS to a glass surface to form the floor. The height (100 μm) and width (800 μm) of the channel were chosen to exert physiological force (wall shear stress <0.3 dyn cm^−2^ or force on BCR–antigen bonds <≈500 pN) and considering clog‐free passage of cells through, a sufficiently long length (10 mm) was chosen to ensure that all the cells contact the bottom surface. The glass surface was functionalized by incubating biotinylated antigen with the surface precoated with NeutrAvidin (Figure 1B, S1A, Supporting Information). When cells are perfused in the device, antigen‐specific cells can bind to the surface (Figure 1C). A COMSOL Multiphysics model simulates cells entering the device and flowing in a horizontal trajectory, as shown with the streamlines (Figure 1D). To interact with the antigen, cells must be close enough to the surface to do so. The model shows the distance along the channel where all the cells in the perfusate settle to the bottom and contact the surface at a given flow (Figure 1E). At 100 μL h^−1^ (or wall shear stress = 0.3 dyn cm^−2^), the highest flow at which we perfused cells in this study, all cells settle to the surface before reaching the outlet in a 10 mm long device.

**Figure 2:**
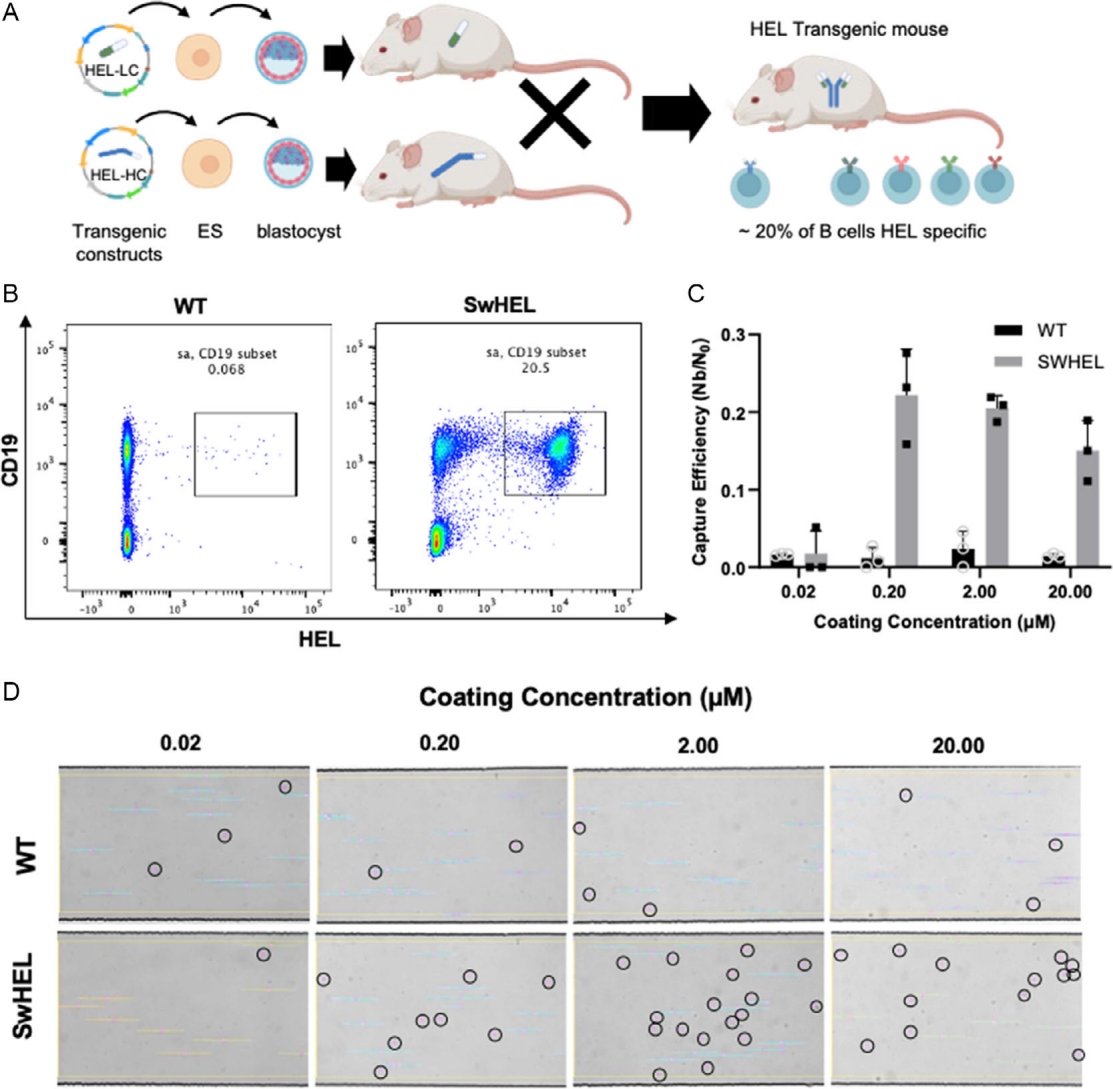
A) Schematic demonstrating how the SwHEL mouse is created with ≈20% of the cells from the spleen at HEL‐specific B cells (produced in part from BioRender). B) Flow cytometry showing subset of spleen cells that are specific to HEL from WT and SwHEL mice. C) Capture efficiency at 100 μL h^−1^ (*n* = 3, mean ± SEM) of WT and SwHEL cells in microfluidic device coated with different concentrations of HEL and D) microscopic imaging showing cells under shear stress of 0.30 dyn cm^−2^ in the device. Circled cells are bound to HEL.

To determine the optimal flow and antigen coating conditions for capturing antigen‐specific B cells, we used high‐affinity HEL‐specific B cells, which were harvested from genetically modified SwHEL mouse spleens (**Figure** **2**A). Roughly 20% of cells in the SwHEL spleen are HEL‐specific B cells, as assessed by flow cytometry conducted prior to each experiment (Figure 2B). We tested various concentrations of HEL coating on the surface. At 0.02 μm, there was no measurable difference between binding of SwHEL cells in comparison to cells from WT mice (Figure 2C). An increase in HEL coating concentration by an order of magnitude resulted in a clear difference in binding between WT and SwHEL cells (Figure 2C), but additional increases in HEL concentration did not further increase cell binding. As such, an antigen coating concentration of 0.2 μm was used for all subsequent studies. Capture efficiency (≈20%) was comparable to flow cytometry data, showing the fraction of positive cells in the WT and SwHEL spleen cell mixtures (Figure 2C). Microscopic imaging illustrates the difference in capture between WT and SwHEL cells at different coating concentrations (Figure 2D). All bound cells were CD19^+^/CD3^−^ consistent with B cells (Figure S1, Supporting Information).

**Figure 3:**
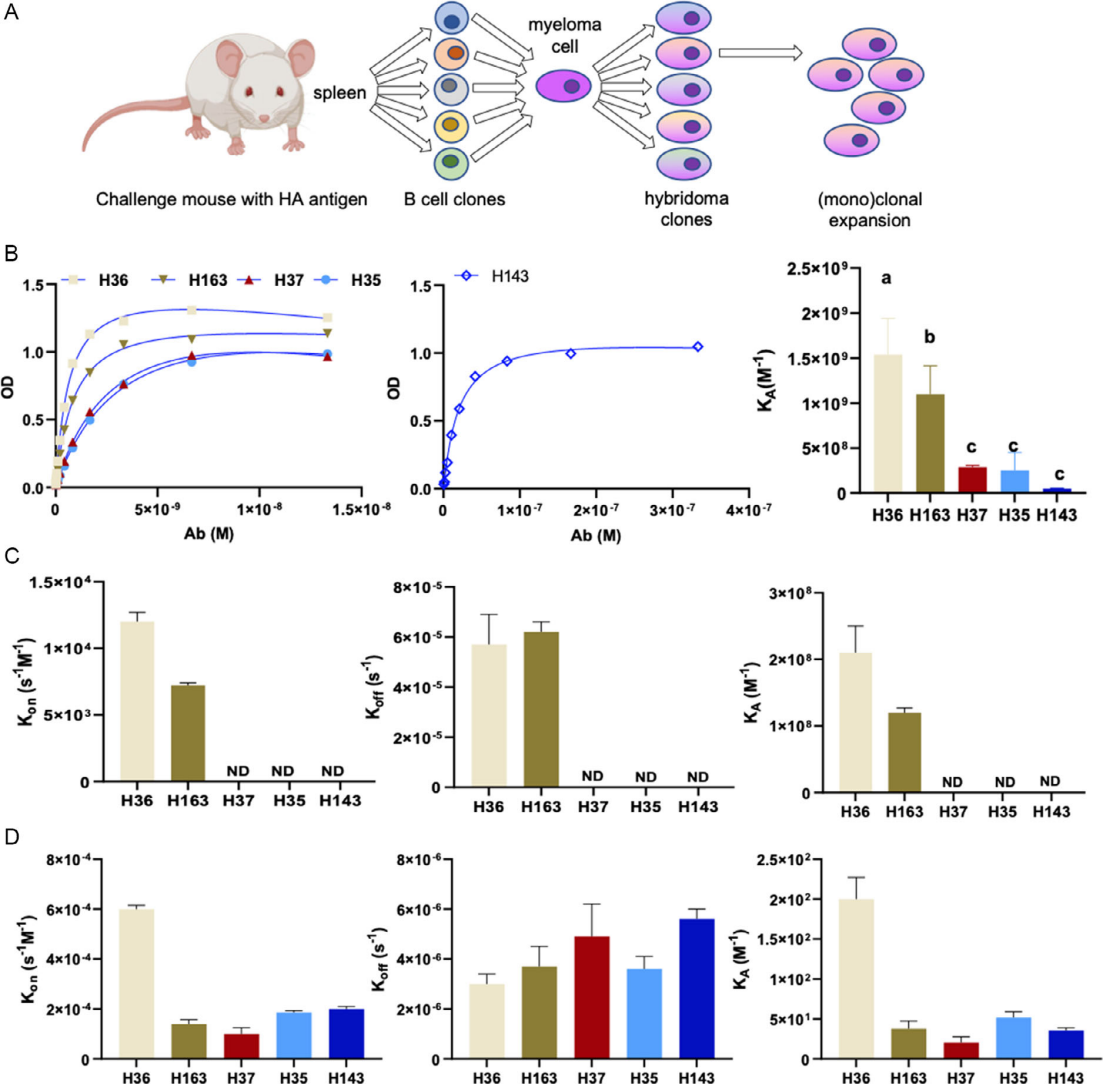
A) Schematic demonstrating how hybridoma technology produces monoclonal hybridoma clones (produced in part from BioRender). B) Optical Density binding curve generated by ELISA assay fitted to measure antibody affinity. Groups with different letters are significantly different (*p* < 0.05). C) Antibody affinity to HA (ND: not detected), and D) avidity to influenza virion measured by OI‐RD.

In peripheral blood of humans vaccinated for or infected by the influenza virus, circulating antibodies have a broad range of binding affinities for hemagglutinin (HA),^[^ ^32^ ^]^ which is the major surface protein of the influenza A virus and is essential to the entry of the virus into host cells. To capture this range of antibody affinities, we used five single‐cell hybridoma clones (**Figure** **3**A) that secrete monoclonal IgG antibodies specific to influenza A/Puerto Rico/8/34 virus HA (Table 1). Before measuring the binding kinetics of the membrane‐bound BCR to HA in the microfluidic device, we first assessed the binding of the secreted monoclonal antibodies (mAbs) to both mammalian‐expressed recombinant HA (affinity) and purified virus particles (avidity) using two well‐established methods: ELISA and oblique‐OI‐RD.^[^ 30, 33 ^]^ For ELISA, antibodies were serially diluted twofold to generate antibody‐binding curves to the virion. The binding curve was then fitted to extract *K*_A_ according to first‐order binding kinetics (Figure 3B). mAb binding was dose‐dependent, with mAb H143 requiring 100‐fold higher starting antibody concentration due to poor binding to the virion compared to the other four mAb tested. The equilibrium affinity constant (*K*_A_, m ^−1^) of the mAbs to the virus particles varied more than 10‐fold from 1 × 10^8^ to 1.5 × 10^9^ m ^−1^ with the mAb H36 showing the highest *K*_A_, followed by mAb H163, H37, H35, and H143 (Figure 3B).

The affinity of the antibodies to HA was also measured by OI‐RD, where the antigen was circulated over fixed antibodies, and then washed away to generate binding curves (Figure S2, Supporting Information). Similar to ELISA, H36 displayed the highest antibody affinity to HA, followed by H163 (Figure 3C). The difference in equilibrium affinity between H36 and H163 was due to a higher *k*_on_ of H36 (Figure 3C). The association and dissociation rates of antibodies from all other cell lines were below the detection threshold. Thus, the antibodies are considered to have low affinity to HA. Avidity measurements were similarly performed by OI‐RD, but with the influenza virion immobilized instead of the HA antigen. While H36 has the highest avidity (highest *k*_on_ and *K*_A_), all antibodies bound to the virion (Figure 3D), further confirming the specificity of the antibodies to HA (Figure 3A,D).

**Figure 4:**
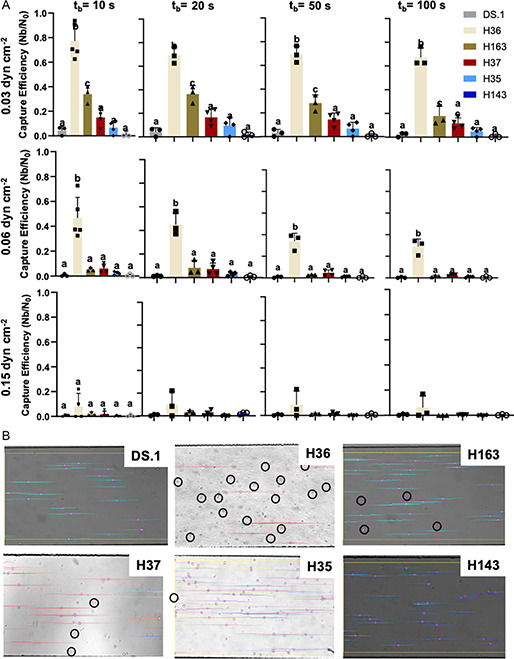
A) Capture efficiency (*n* ≥ 3, ± SEM) of five HA‐specific hybridoma lines and negative control under shear stress of 0.03, 0.06, and 0.15 dyn cm^−2^ with minimum binding criteria of 10, 20, 50, and 100 s. Groups with different letters are significantly different (*p* < 0.05). B) Microscopic imaging showing cells under shear stress of 0.06 dyn cm^−2^ in the device. Circled cells are bound to HA.

To observe how B cells (i.e., the membrane‐bound BCR) bind to HA in the device under force, cells from the five hybridoma lines and DS.1 (negative control) were perfused into a laminar microfluidic channel coated with HA (0.2 μm) under a range of constant shear force at the surface (0.03 − 0.15 dyne cm^−2^). Cells were perfused in the device at three flow rates (10, 20, and 50 μL h^−1^), corresponding to three shear stresses at the surface (0.03, 0.06, and 0.15 dyne cm^−2^). These fluid flows are expected to apply 65, 130, and 325 pN forces which are in the physiological range of forces experienced by BCR–antigens bonds.^[^ ^10^ ^]^ The binding criterion was set to a minimum bound lifetime of 10, 20, 50, or 100 s. For all binding criteria, H36 demonstrated the highest capture efficiency (**Figure** **4**A,B) ranging from as high as 0.78 (78%) at the lowest shear (0.03 dyne cm^−2^) and lowest minimum binding criteria (10 s), to as low as 0.08 (8%) at the highest shear (0.15 dyne cm^−2^) and highest minimum binding criteria (100 s). At the two higher shear rates, the other cell lines showed little binding in the device, regardless of binding criteria. At the lowest shear and all minimum binding criteria, H36 was followed by H163, H37, and H35 with H143 showing little‐to‐no binding.

**Figure 5:**
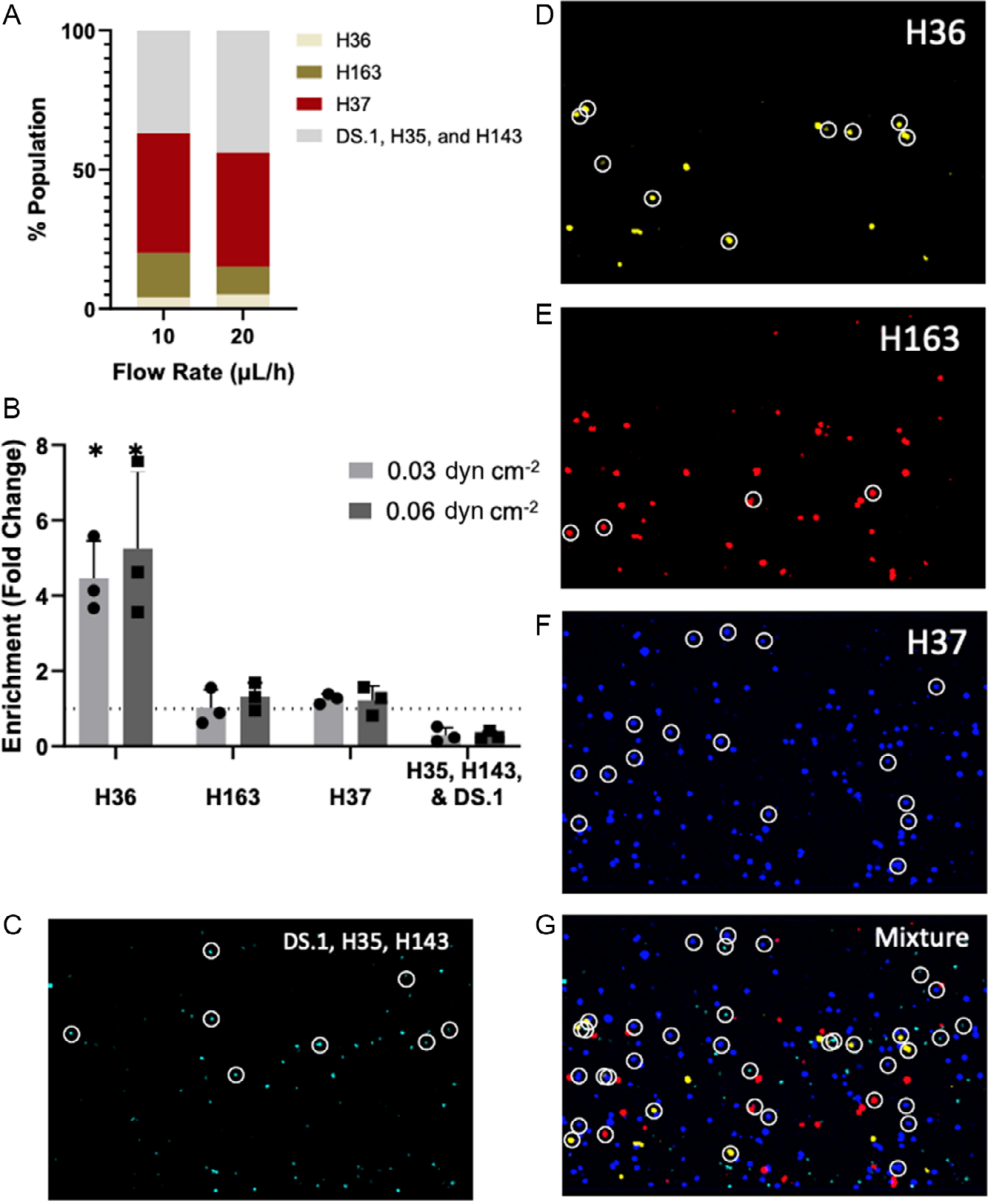
A) Composition of hybridoma cell lines in the mixture that is perfused in the device. B) Fold change in enrichment (Bound Cells (%)/Perfusate (%)) for different cell populations in mixture when perfused at 10 and 20 μL h^−1^ (*n* = 3, mean ± SEM). *significantly different from other groups (*p* < 0.05). C–G) Microscopic imaging showing cells under shear stress of 0.06 dyn cm^−2^. H36 (yellow), H163 (red), H37 (blue), and DS.1, H35 and H143 (cyan) are shown individually and in the mixture in which they were perfused. Circled cells are bound to HA. Bound cells were measured according to a minimum binding criterion of 10 s. Values that differ from 1‐fold change with statistical significance (*p* < 0.05) are indicated.

The higher capture efficiency of H36 provides an opportunity to use the microfluidic device to enrich (or capture) a mixed population of hybridoma clones in H36. We created a mixed population of the five hybridoma cell lines as well as DS.1, with the concentration of H36 set at <5% representing a relatively dilute or rare cell (**Figure** **5**A). The three highest binding clones were fluorescently labeled with different colors and the remaining three cell lines labeled with a fourth color. The mixed cell population was introduced into the device at two flows (10 and 20 μL h^−1^) corresponding to the two lowest shear (0.03 and 0.06 dyne cm^−2^). Over 20% of the cells captured on the device (Figure 5B–G) were H36, representing a 4‐to‐5‐fold enrichment. H163 and H37 were neither enriched nor diluted on the surface, whereas the remaining three cell lines were diluted.

**Figure 6:**
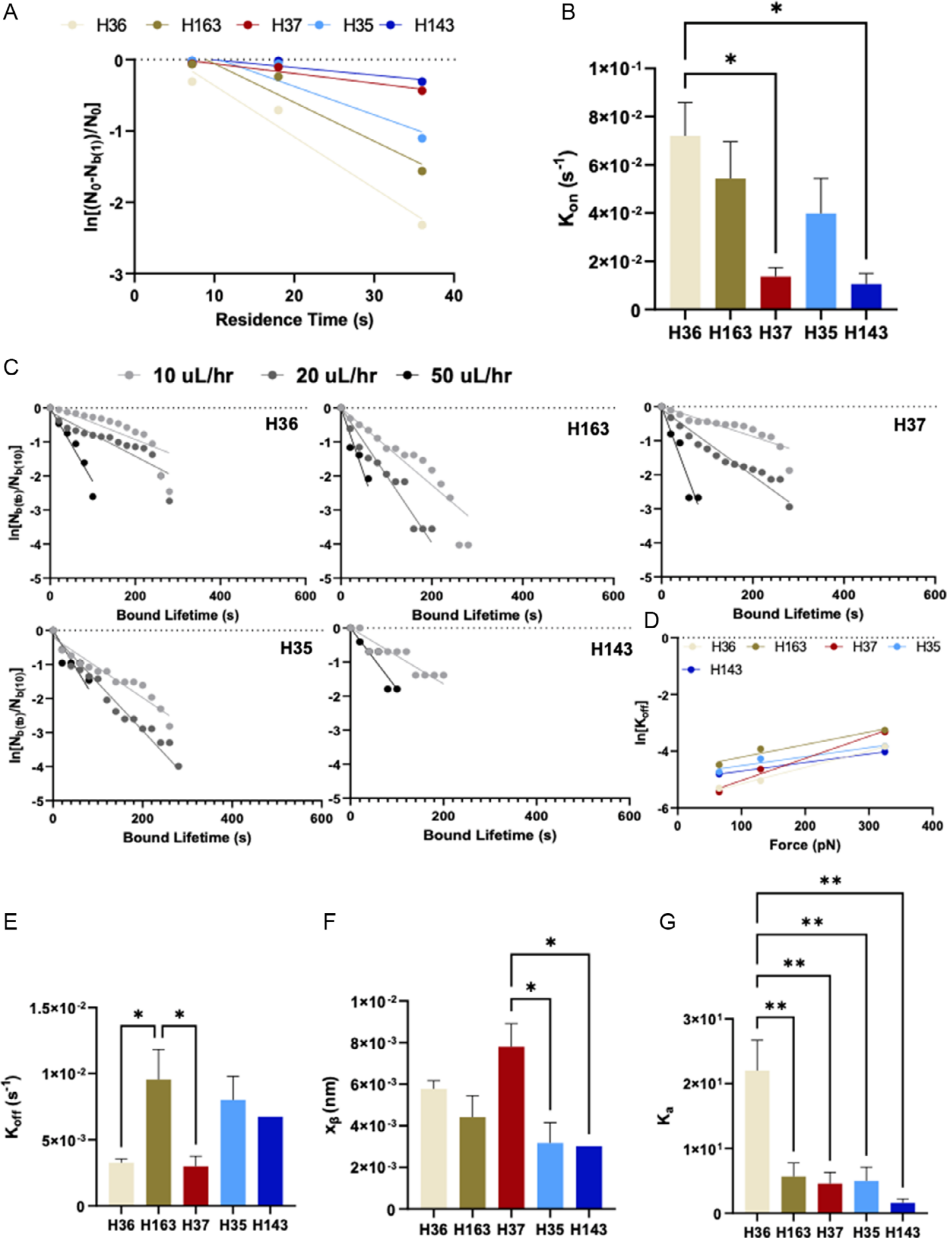
A) Data fit based on first‐order kinetic model to measure effective *k*_on_. B) Measured values of effective *k*_on_ for the different hybridoma cell lines. C) Survival curve fits to determine force‐dependent *k*_off_, where bound lifetime is the length of time the cells remain bound for more than 10 s and D) data fit to determine reactive compliance (*x*_*β*_) and effective *k*_off_ at zero‐force. E) Values of effective *k*_off_ at zero‐force and F) reactive compliance (*x*_*β*_). G) Membrane‐bound BCR affinity (effective $\frac{k_{\text{on}}}{k_{\text{off}}}$) to HA. *significantly different (*p* < 0.05); **significantly different (*p* < 0.01).

BCR–antigen kinetic parameters are generally measured using the secreted antibodies. Here, we used membrane‐bound BCR binding to HA under force to measure the reactive compliance (*x*_*β*_) of the intermolecular bonds and effective *k*_on_ and *k*_off_. The force‐dependent *k*_off_ is related to the force independent $k_{\text{off }}^{0}$ by the Bell model,^[^ ^28^ ^]^ $k_{\text{off}}=k_{\text{off}}^{0}e^{x_{\beta }F/K_{B}T}$, where *F* is the force applied on the bond, and *K*_B_ is Boltzmann's constant. A more detailed theoretical explanation of these parameters is provided elsewhere.^[^ ^34^ ^]^ Briefly, the $k_{\text{off }}^{0}$ is intrinsic off‐rate of the bond between the BCR and antigen and the reactive compliance is a parameter that accounts for the fact that the BCR is not a free‐floating solute but is anchored to the elastic membrane of the cells. This equation shows that increase in the force (*F*) can exponentially increase the *k*_off_. *k*_on_, a first‐order kinetic model was derived to fit cell binding data that depend on the number cells that experience an initial tethering of at least 1 s to the surface (**Figure** **6**A). Effective *k*_on_ measurements for H143 and H35 were significantly lower than H36, H163, and H37 (Figure 6B). To measure effective *k*_off_ and *x*_*β*_, force‐dependent survival curves were generated for cells with minimum binding criteria of 10 s (Figure 6C). Force‐dependent values of *k*_off_ were then fitted to determine *x*_*β*_. The fit was extrapolated to zero force to determine effective *k*_off_ (Figure 6D). Effective *k*_off_ is lowest for H36 and H37. While H163 has a *k*_on_ similar to that of H36, its *k*_off_ is significantly higher (Figure 6E). Additionally, *x*_*β*_ measurements show that not all hybridoma cells behave the same when subjected to shear force in the device, with H35 and H143 having lower *x*_*β*_ compared to H37, consistent with off‐rates that are less sensitive to force (Figure 6F). Finally, the affinity constant, the ratio of the effective on‐ and off‐rates, shows that H36 has the highest affinity to HA (Figure 6G). Comparing these measurements in the device to the affinity measurements by ELISA and OI‐RD shows a strong correlation between all three assays (Figure S3, Supporting Information).

## 4 Discussion

Recent efforts to optimize the isolation of high‐affinity antibodies for therapeutic applications or to assess the functional immune status of individuals have highlighted the importance of efficient screening and characterization of B cells and their antibodies.^[^ 35, 36, 37, 38, 39, 40, 41 ^]^ These methods rely on equilibrium binding properties of BCR/antibody–antigen bonds, followed by cumbersome and expensive steps of cloning, expression, and mAb purification. Using a very simple microfluidic device (single‐rectangular channel), we demonstrate that by flowing B cells at a controlled shear over a surface functionalized with antigen, we can efficiently capture and enrich a population of antigen‐specific high‐affinity B cells. Furthermore, this methodology can also be used to assess kinetic binding parameters of the membrane‐bound BCR (*k*_on_, *k*_off_, *K*_A_, and *x*_*β*_). We found that *K*_A_ correlated well with traditional assays that measure the equilibrium binding affinity of the secreted antibody. Our results demonstrate that a simple microfluidic strategy can efficiently identify antigen‐specific, high‐affinity, rare B cells from a larger population and thus could be used as a cost‐effective strategy to identify B cell clones generating mAb for a host of clinical applications or to assess the functional immune status of an individual in a timely fashion.

The creation of antigen‐specific high‐affinity mAbs has a host of important clinical applications including the treatment of patients with viral infections (most recently COVID‐19^[^ ^42^ ^]^), allergic inflammation in diseases such as asthma,^[^ ^43^ ^]^ autoimmune diseases such as rheumatoid arthritis,^[^ ^44^ ^]^ and cancer.^[^ ^45^ ^]^ All told, the annual global sales of therapeutic and prophylactic mAbs are in excess of US$75 billion.^[^ ^46^ ^]^ The process to create mAbs utilizes either hybridoma technology or antibody phage display.^[^ ^47^ ^]^ Although generally effective, both techniques have significant drawbacks, including the cost and overall time (months to years once an antigen has been identified) to create the mAb.^[^ ^48^ ^]^ This is particularly important for creation of mAbs for diseases with a rapidly changing antigen landscape such as during infections with highly mutating RNA viruses (e.g., omicron variant of SARS‐CoV2^[^ ^49^ ^]^) and most solid cancers.^[^ ^50^ ^]^ A key result of our study is the high correlation between the avidity of the membrane‐bound BCR measured in our microfluidic device and the affinity of the secreted antibodies measured by ELISA and OI‐RD. As such, our microfluidic strategy, which employs tunable force‐dependent (shear stress) binding, can be used to capture and enrich very‐high‐affinity B cell clones from a mixed population and thus the sources of antigen‐specific high affinity mAbs. Using only a single pass at a single flow (shear stress), we enriched a dilute (<5%) population of high‐affinity B cells by 4‐to‐5 fold. One can then easily imagine repeating this process to further enrich by altering the flow to capture additional populations of B cells of defined affinity. The flow‐based microfluidic approaches have been used for selecting cancer cells using antibodies^[^ ^51^ ^]^ and antigen‐specific T‐cells using whole tumor cells.^[^ ^52^ ^]^ This literature provides a foundation for the antigen‐based separation technology presented in our study.

The distinct advantage of our approach over current methods used in B‐cell characterization is it obviates the laborious steps of antibody testing and screening because the force‐dependent selection of the high‐affinity B cell clone has already performed this task. It is noteworthy that our method cannot distinguish between a small number of high‐affinity interactions and a large number of low‐affinity interactions; however, the controlled interaction between the B cell and surface in laminar flow creates an environment consistent with a similar number of BCRs interacting with the surface for each cell.

An alternative to our microfluidic strategy to identify antigen‐specific, high‐affinity B cell clones is to utilize affinity maturation.^[^ ^53^ ^]^ Following exposure to a pathogen, B cells undergo affinity maturation in which somatic hypermutation creates BCRs with higher antigen affinity and thus improved immune response to a specific antigen. Affinity maturation creates a diverse population of B cells with frequently changing characteristics.^[^ ^54^ ^]^ Although BCR affinity is thought to predict functional immunity,^[^ ^4^ ^]^ what features of the diverse population of B cells are predictive is not known. For example, does the high‐affinity B cells predict functional immunity? If so, how high is “high”? Alternatively, a population of lower‐affinity B cells may be adequate. If this is the case, what range of affinity is required? By demonstrating that our technology can capture and enrich a high affinity B cell subpopulation, it is easy to extrapolate how the technology could be used to quantify the full spectrum of B cell binding affinity to a target antigen. For example, the first step (high shear and potentially multiple passes) captures the highest‐affinity B cells in the device, which can then easily be removed and collected. The population of B cells which remain (unbound from the first pass) are collected and passed through the device at a lower shear. The next highest‐affinity B cells attach to the surface, which can then be collected. The cells which did not bind are collected and the process repeated at a lower shear. Alternatively, a single long channel could be designed with progressively a larger cross‐sectional area, each area presenting a lower shear force and capturing a different population of B cells. In either strategy, a series of B cell populations are collected and quantified in a progressively descending order of affinity. Although most of our studies were generated using B cell lines, in proof‐of‐concept studies with primary spleen cells from SwHEL BCR transgenic mice, expressing a BCR of known high affinity for HEL,^[^ ^17^ ^]^ we demonstrate that our device also allows study of primary cells. Assessing a population of B cells quantitatively and at an individual level reliably and affordably could be instrumental in pandemic responses. For example, rapidly assessing the spectrum of B cell binding affinity at a point in time could provide a surrogate for the immune status of individuals and thus provide important information for the strategic distribution of limited resources (drug and vaccine).

In summary, we present a simple microfluidic strategy that utilizes shear stress (force) to characterize the force‐dependent antigen‐specific binding characteristics (*k*_on_, *k*_off_, *K*_A_, and *x*_*β*_) of the membrane‐bound BCR. We show that the binding affinity of five hybridoma cell lines specific to influenza HA is remarkably variable but that the affinity of the cell membrane‐bound BCR correlates well with the binding affinity of the secreted antibodies. The technology can be used to easily capture and enrich a population of antigen‐specific, high‐affinity B cells and thus be used to quantitatively characterize the full spectrum of binding affinity for a diverse population of B cells. The technology is easily scalable and thus has potentially important applications to simplify and reduce the cost and time to create mAb, as well as to rapidly and cost‐effectively assess the spectrum of B cell affinity and thus the functional immune status of an individual.

## Conflict of Interest

The authors declare no conflict of interest.

## Supporting information

Supplementary Material

## Data Availability

The data that support the findings of this study are available from the corresponding author upon reasonable request.
