# Supplementary material for: A Microfluidic Strategy to Capture Antigen‐Specific High‐Affinity B Cells
Source: Adv Nanobiomed Res. 2024 Apr 26;4(6):2300101. doi: 10.1002/anbr.202300101 (PMC12203763; doi:10.1002/anbr.202300101)
Supplement: Supplementary file 1 — Supplementary Material [file ANBR-4-0-s001.pdf]

SUPPLEMENTAL INFORMATION

A microfluidic strategy to capture antigen-specific high affinity B cells

Ahmed M. Alhassan[1,^], Venktesh S. Shirure[1,^], Jean Luo[2], Bryan B. Nguyen[1], Zachary A. Rollins[1], Bhupinder S. Shergill[1], Xiangdong Zhu[3,#], Nicole Baumgarth[2,4,#], and Steven C. George[1,#,]*

[1]Department of Biomedical Engineering, University of California, Davis.

[2]Department of Pathology, Microbiology, and Immunology, University of California, Davis.

[3]Department of Physics and Astronomy, University of California, Davis.

[4]Department of Molecular Microbiology and Immunology, Bloomberg School of Public Health and Department of Molecular and Comparative Pathobiology, School of Medicine, Johns Hopkins University, Baltimore, MD

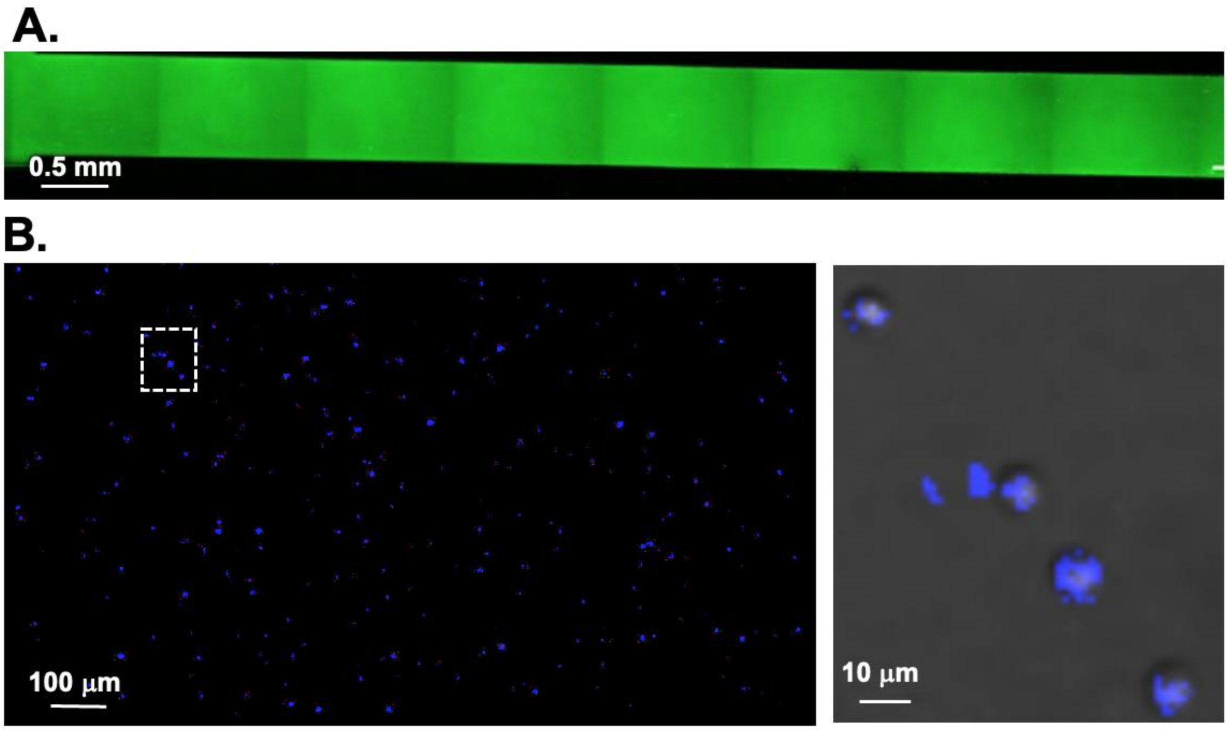

Figure S1. Biotin-FITC bound to NeutrAvidin coated device (A). CD19 coated SwHEL cells bound to HEL in device (B).

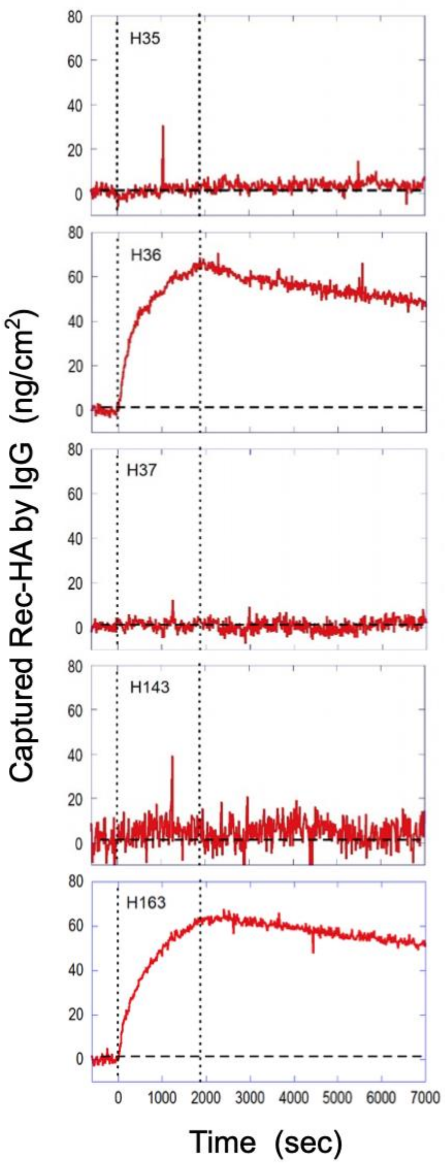

Figure S2. Binding curves of Recombinant HA (Rec-HA) to H35, H36, H37, H143, and H163 using an OI-RD detected, microarray-based assay system. Two vertical dotted lines mark starts of association phase and dissociation phase of the affinity assay. The concentration of the recombinant HA is 300 nM. The curves are fit to a 1-to-1 Langmuir reaction model to yield association rate constants k_{on} ($M^{-1}sec^{-1}$) and dissociation rate constants k_{off} (sec^{-1}). Affinity constants K_a of Rec-HA to IgG are determined from $K_a = k_{on} / k_{off}$.

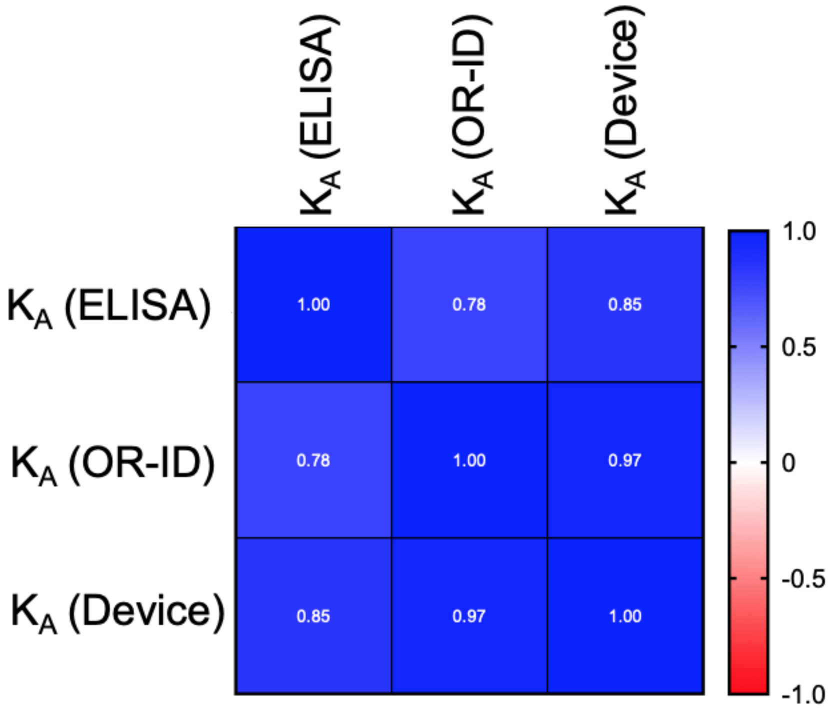

Figure S3. Pearson correlation matrix of K_A values measured by three different assays: ELISA, OI-RD and microfluidic device.
